# An automatic hypothesis generation for plausible linkage between xanthium and diabetes

**DOI:** 10.1038/s41598-022-20752-0

**Published:** 2022-10-20

**Authors:** Arida Ferti Syafiandini, Gyuri Song, Yuri Ahn, Heeyoung Kim, Min Song

**Affiliations:** Department of Library and Information Science, Yonsei University, Seoul, Republic of Korea

**Keywords:** Computational science, Scientific data

## Abstract

There has been a significant increase in text mining implementation for biomedical literature in recent years. Previous studies introduced the implementation of text mining and literature-based discovery to generate hypotheses of potential candidates for drug development. By conducting a hypothesis-generation step and using evidence from published journal articles or proceedings, previous studies have managed to reduce experimental time and costs. First, we applied the closed discovery approach from Swanson’s ABC model to collect publications related to 36 Xanthium compounds or diabetes. Second, we extracted biomedical entities and relations using a knowledge extraction engine, the Public Knowledge Discovery Engine for Java or PKDE4J. Third, we built a knowledge graph using the obtained bio entities and relations and then generated paths with Xanthium compounds as source nodes and diabetes as the target node. Lastly, we employed graph embeddings to rank each path and evaluated the results based on domain experts’ opinions and literature. Among 36 Xanthium compounds, 35 had direct paths to five diabetes-related nodes. We ranked 2,740,314 paths in total between 35 Xanthium compounds and three diabetes-related phrases: type 1 diabetes, type 2 diabetes, and diabetes mellitus. Based on the top five percentile paths, we concluded that adenosine, choline, beta-sitosterol, rhamnose, and scopoletin were potential candidates for diabetes drug development using natural products. Our framework for hypothesis generation employs a closed discovery from Swanson’s ABC model that has proven very helpful in discovering biological linkages between bio entities. The PKDE4J tools we used to capture bio entities from our document collection could label entities into five categories: genes, compounds, phenotypes, biological processes, and molecular functions. Using the BioPREP model, we managed to interpret the semantic relatedness between two nodes and provided paths containing valuable hypotheses. Lastly, using a graph-embedding algorithm in our path-ranking analysis, we exploited the semantic relatedness while preserving the graph structure properties.

## Introduction

Drug development is both expensive and time-consuming. Therefore, many studies have focused on reducing the time and costs of drug development. Multidisciplinary approaches and the implementation of computational methods are strongly encouraged to reduce the workload in drug development. Previous studies have applied artificial intelligence approaches to help reduce drug development costs^1,2^. As the quantity of biomedical literature has increased, there has been steadfast interest in applying text-mining techniques and Literature-based Discovery (LBD) to generate applicable drug compound candidates^3^. We can extract information from biomedical literature and generate facts using text-mining techniques. Then, we can employ the LBD concept to generate hypotheses for drug development using those facts. Analyzing existing facts garnered from biomedical literature to generate new hypotheses is called “Conceptual Biology”^4^.

Previous works suggest that combining LBD and text-mining techniques to generate hypotheses for drug development can significantly decrease the experiment time and cost^5–8^. However, despite the significant growth of studies in this field, hypothesis generation for drug development purposes remains challenging. The high dimensionality of biological substances and the number of related publications can be a significant obstacle to discovering possible linkages between entities^9^. Moreover, the number of generated paths during hypothesis generation is relatively high, making it difficult to gain insights. Therefore, to tackle such problems, this paper proposed a complete framework for hypothesis generation utilizing LBD and text-mining techniques with additional path-ranking steps to select critical paths and recommended them for further experiments in drug development.

We investigated the natural compounds found in Xanthium and their connectedness with diabetes as a case study. Those compounds are extracted from medicinal plants in the *Xanthium* genus such as *Xanthium strumarium*^10^ and *Xanthium sibiricum*^11^. A previous study found that *Xanthium strumarium* might have an anti-diabetic effect because its fruit reduced the elevation of plasma glucose levels in diabetic rats^12^. Another study found that *Xanthium sibiricum* Patrin ex Widder water extracts (CEW) could increase the sugar tolerance in normal mice and decrease the blood sugar level in diabetic mice^13^. Further experiments^14^ also proved that Xanthium compounds and diabetes are significantly related but there have been few studies about the complete biological interactions between these two. In addition, we found that diabetes is one of the most common endocrine disorders with a high probability of severe complications^15,16^. Diabetes is also a lifelong disease with no available cure. Several synthetic drugs are available for diabetes treatments but they are costly, have many side effects, and are unsuitable for long-term consumption^17^. Therefore, there is an urgent need to find natural compounds for long-term diabetes treatment.

To generate hypotheses for Xanthium compounds and diabetes, we applied Swanson’s ABC model^18^, which is a known LBD model for bio-literature mining. This model has two main steps we need to execute, constructing a knowledge base and generating paths. We utilized the PKDE4J tool^19^ and BioPREP model^20^ to extract entities and relations from retrieved PubMed articles and construct the knowledge base. PKDE4J is a dictionary-based Named Entity Recognition (NER) and rule-based relation extraction tool, while BioPREP is a pre-trained language model specifically built for learning biomedical text. The BioPREP model learned sentences, transformed them into embedding representations, and forwarded them for predicate (relation) classification. We generated simple paths from our knowledge base and ranked them using our proposed path-ranking algorithm, which combines the graph-embedding approach^21^ and the encoder–decoder architecture. We relied on a literature-based study and experts’ opinions to validate our path-ranking results.

We highlighted our contributions in this paper: constructing a Xanthium compounds-diabetes knowledge base, proposing a path-ranking approach using graph-embedding values, and generating hypotheses for drug development experiments using Xanthium compounds.

## Related works

Swanson first implemented a literature-based discovery approach to investigate linkages between dietary fish oil and Raynaud’s syndrome^18^. This approach is known as the ABC model and pioneered biomedical literature mining. Swanson’s ABC model generated constructive hypotheses and was helpful for further investigation. With the growing number of publications and digitalization, more studies have applied and co-opted Swanson’s ABC model with text-mining techniques for knowledge discovery and hypothesis generation^22^. We can cover more extensive collections with text-mining techniques and significantly reduce analysis bias. Moreover, we increased the probability of discovering new biological concepts and produced more compact hypotheses for drug development^23–25^.

The basic principle in the ABC model is constructing a knowledge base (usually represented as a graph) using open or closed discovery approaches. Essentially, an open discovery aimed to discover C instances given the A and B instances, while closed discovery aimed to discover B instances given the A and C instances. We can directly observe and choose the A, B, or C instances in small collection cases. Nevertheless, we need to use an automated approach to identify those instances for significant collection cases. PKDE4J^19^—a dictionary-based tool for entity recognition and relation extraction—is one solution for processing large-scale data collections. PKDE4J can automatically identify entities and label the relationships between two entities in sentences. For entity extraction, PKDE4J utilized multiple dictionaries with a vast vocabulary. In a previous evaluation, PKDE4J outperformed several machine learning-based tools—including Neji^26^—in the NER task. PKDE4J gave better performance, especially for matching and labeling bio entities with multiple terms.

PKDE4J employed a rule-based approach for relation extraction, which might be powerful but may not cover all conditions. Other than rule-based approaches, previous studies proposed supervised approaches that utilize neural network structures to extract relation information from texts^27–29^. However, those methods were less efficient because they required determining features beforehand. The development of a pre-trained language model such as BERT^30^ has enabled the processing of texts without additional feature-processing steps. BERT employs bidirectional encoders that learn sentences and passages in a contextual manner. We can fine-tune BERT for specific vocabularies and collections such as biomedical literature; BioPREP is one of several BERT models explicitly trained for biomedicine^20^. BioPREP fine-tuned the previously available BERT models SciBERT^31^ and BioBERT^32^ using SemMedDB^33^. SemMedDB is a publicly available large dataset for biomedical entity and relation extraction. Fine-tuning a language model with SemMedDB can tackle the coverage problem when building a relation-extraction model.

Once we finish the knowledge base construction, we need to generate paths and conclude hypotheses based on those paths. Depending on the knowledge base size and path depths, the number of generated paths could be enormous and analyzing them individually would be excessive. Therefore, we need an automated approach such as a path-ranking algorithm (PRA). A PRA would help identify critical paths for hypothesis generation and has emerged as a promising method for learning inference paths in large knowledge graphs^34^. The most common step in PRA is calculating the triple score (node–relation–node) and calculating the path score. A previous study^35^ proposed a triple score calculation using semantic relatedness between nodes and compared their approaches with baseline approaches, such as co-occurrence, word embedding, COALS, and random index. They concluded that their approach performed well compared to those baseline approaches. Despite its effectiveness, their approach depended on the quantity of collected data and was not suitable for handling networks with multiple relations. Therefore, this paper proposed a PRA algorithm that employed a graph-embedding approach called Complex^21^ to calculate a triple score. The Complex algorithm considers relation information in edges and maps them into complex space. Using this algorithm, we can obtain embedding values that reflect on multiple relation conditions and the importance of triples.

Previous studies have developed various LBD tools for generating hypotheses to support drug discovery. One early tool in LBD, Swanson’s Arrowsmith, utilized the term co-occurrence to identify associations between entities^36^. Other tools such as BITOLA^37^, DAD^38^, LitLinker^39^, Manjal^40^, and LION^41^ provide similar LBD functions focusing on biomedical literature mining. The success of hypothesis generation using the LBD approach significantly depended on path selection and scoring efficiency. Previous studies attempted to use various representation models to calculate path scores and filter paths based on those scores. Despite numerous advantages in implementing a graph-embedding algorithm on heterogeneous networks (knowledge bases)^42^, it has not been widely implemented in the LBD framework.

## Data

Our hypothesis generation framework followed the close discovery approach of Swanson’s ABC model^22^. The close discovery approach tried to identify B entities that connected the A entity to the C entity. Both A and C entities were known entities that we can use as source and tail nodes in path retrieval. This paper defined A entities as Xanthium compounds and C entities as diabetes-related terms or phrases. Since we aimed to discover B entities (multiple types of bio entities) that connected those entities, we formulated search queries using Xanthium compounds and diabetes to retrieve documents from PubMed.

Previous studies^10,43^ discovered 243 compounds from Xanthium, only 36 of which were closely related to diabetes. Therefore, we used those 36 compounds to retrieve titles and abstracts from PubMed in our search queries. We retrieved documents from PubMed using queries from Table 1 in January 2021 and collected 805,839 titles and abstracts. After pre-processing and duplicate removal, 763,155 titles and abstracts remained in our collection. Then, we tokenized each sentence from abstracts and titles and used them for the NER and relation-extraction tasks. We provided a document sample related to 4,5-dicaffeoylquinic acid in Table 2.

**Table 1:** Search queries for document retrieval.

“A” node (Xanthium compounds)	“C” nodes (diabetes)
1,3-di-O-caffeoylquinic acid[TIAB] OR 2-acetolactate[TIAB] OR acetone[TIAB] OR adenosine[TIAB] OR alkaloids[TIAB] OR aloe emodin[TIAB] OR atractyloside[TIAB] OR balanophonin[TIAB] OR beta-sitostenone[TIAB] OR beta-sitosterol[TIAB] OR betulin[TIAB] OR betulinic acid[TIAB] OR caffeic acid[TIAB] OR caffeic acid ethyl ester[TIAB] OR campesterol[TIAB] OR chlorogenic acid[TIAB] OR choline[TIAB] OR emodin[TIAB] OR ergosterol[TIAB] OR quercetin[TIAB] OR rhamnose[TIAB] OR scopoletin[TIAB] OR stigmasterol[TIAB] OR syringaresinol[TIAB] OR thiourea[TIAB] OR water-soluble glycosides[TIAB] OR 3,5-dicaffeoylquinic acid[TIAB] OR 4,5-dicaffeoylquinic acid[TIAB] OR ferulic acid[TIAB] OR formononetin[TIAB] OR hexadecanoic acid[TIAB] OR N-trans-feruloyl tyramine[TIAB] OR oleanolic acid[TIAB] OR oleic acid[TIAB] OR ononin[TIAB] OR protocatechuic acid[TIAB]	diabet*[TIAB] ([TIAB] retrieving articles that contain a certain keyword in titles or abstracts) OR diabetes[MH] ([MH] retrieving articles that discuss diabetes in the MeSH list)

**Table 2:** Document sample.

**Title:** The anti-inflammatory activities of Ainsliaea fragrans Champ. extract and its components in lipopolysaccharide-stimulated RAW264.7 macrophages through inhibition of NF-κB pathway
**Journal:** Journal of ethnopharmacology
**Abstract:** The anti-inflammatory activities of Ainsliaea fragrans Champ. Extract and its components in lipopolysaccharide-stimulated RAW264.7 macrophages through inhibition of NF-κB pathway. Ainsliaea fragrans Champ. (A. fragrans) is a traditional Chinese herbal that contains components like 3,5-dicaffeoylquinic acid and **4,5-dicaffeoylquinic acid**. It exhibits anti-inflammatory activities which has been used for the treatment of gynecological diseases for many years in China. The aims of the present study were to investigate the anti-inflammatory activities of A. fragrans and elucidate the underlying mechanisms with regard to its molecular basis of action for the best component. The anti-inflammatory effects of A. fragrans were studied by using lipopolysaccharide (LPS)-stimulated activation of nitric oxide (NO) in mouse RAW264.7 macrophages. Expression of inducible NO synthase (iNOS) and pro-inflammatory cytokines, inhibitory κBα (IκBα) degradation and nuclear translocation of NF-κB p65 were further investigated. The present study demonstrated that A. fragrans could suppress the production of NO in LPS-stimulated RAW264.7 macrophages. Further investigations showed A. fragrans could suppress iNOS expression. A. fragrans also inhibited the expression of tumor necrosis factor-alpha and interleukin-6. A. fragrans significantly decreased the degradation of IκBα, reduced the level of nuclear translocation of p65. All these results suggested the inhibitory effects of A. fragrans on the production of inflammatory mediators through the inhibition of the NF-κB activation pathway. Our results indicated that A. fragrans inhibited inflammatory events and iNOS expression in LPS-stimulated RAW264.7 cells through the inactivation of NF-κB pathway. This study gives scientific evidence that validate the use of A. fragrans in treatment of patients with gynecological diseases in clinical practice in traditional Chinese medicine

## Methods

### Knowledge base construction

**Figure 1:**
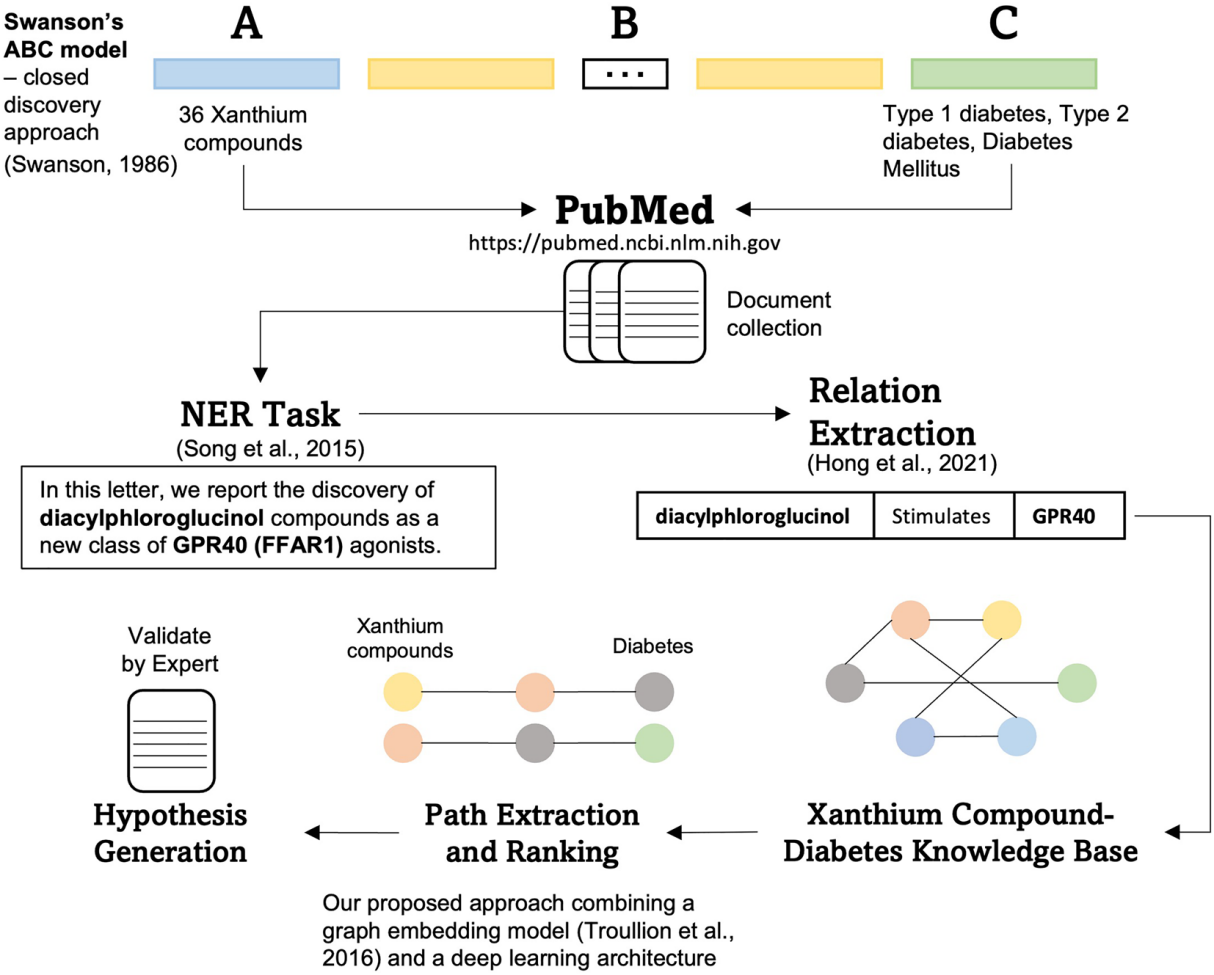
Our research framework.

We extracted bio entities and relations from our document collection to construct a knowledge base (graph) for hypothesis generation. There are two steps in our knowledge base construction: entity extraction (NER task) and relation extraction. Figure 1 illustrates the complete flow of our research.

### Entity extraction (NER task)

To extract bio entities from our document collection, we used a knowledge extraction engine called PKDE4J^19^. This tool has a dictionary-based NER module where we can use custom dictionaries depending on which entities we want to extract. We decided to use eight bio entities related to drug development: genes (including protein and RNA), compounds (including Xanthium compounds), phenotypes, biological processes, and molecular functions. In addition, we utilized five different dictionaries from several biological databases, as described in Table 3.

**Table 3:** Dictionary summary.

	Dictionary	Sources	Number of Entities
1	Gene	Entrez^44^, Ensembl^45^, BioGrid^46^, PharmGKB^47^, UniProt ID^48^, NCBI taxonomy^49^	20,503,546
2	Compound	PubChem^50^, ChEMBL^51^, ChEBI^52^, CAS^53^, BindingDB^54^, KEGG^55^, DrugBank^56^	64,966,141
3	Phenotype	Medical Subject Headings (MeSH)	109,062
4	Biological Process	Gene Ontology^57^	30,492
5	Molecular Function	Gene Ontology^57^	12,257

As mentioned in the data section, we used the [MH] code for our document retrieval. Hence, during the retrieval process, not only were “diabetes”-related documents retrieved, we also retrieved documents related to “diabetes mellitus,” “type 1 diabetes,” “type 2 diabetes,” “gestational diabetes,” and “pre-diabetes.” This paper analyzed every possible hypothesis (path) between Xanthium compounds and those five diabetes-related phrases.

### Relation extraction

For relation extraction, we examined every sentence in our document collection. If there were two or more unique bio entities in a sentence, we proceeded with the relation-extraction step using a pre-trained model called BioPREP^20^. BioPREP employs a BioBERT-based model that it fine-tunes using the SemMedDB dataset^33^. Using the BioPREP model, we extracted 28 relations, namely: “process of,” “part of,” “location of,” “diagnoses,” “interacts with,” “treats,” “coexists with,” “is a,” “uses,” “precedes,” “associated with,” “causes,” “affects,” “administered to,” “disrupts,” “occurs in,” “complicates,” “inhibits,” “stimulates,” “augments,” “compared with,” “prevents,” “method of,” “neg interacts with,” “neg affects,” “produces,” “manifestation of,” and “higher than.”

**Figure 2:**
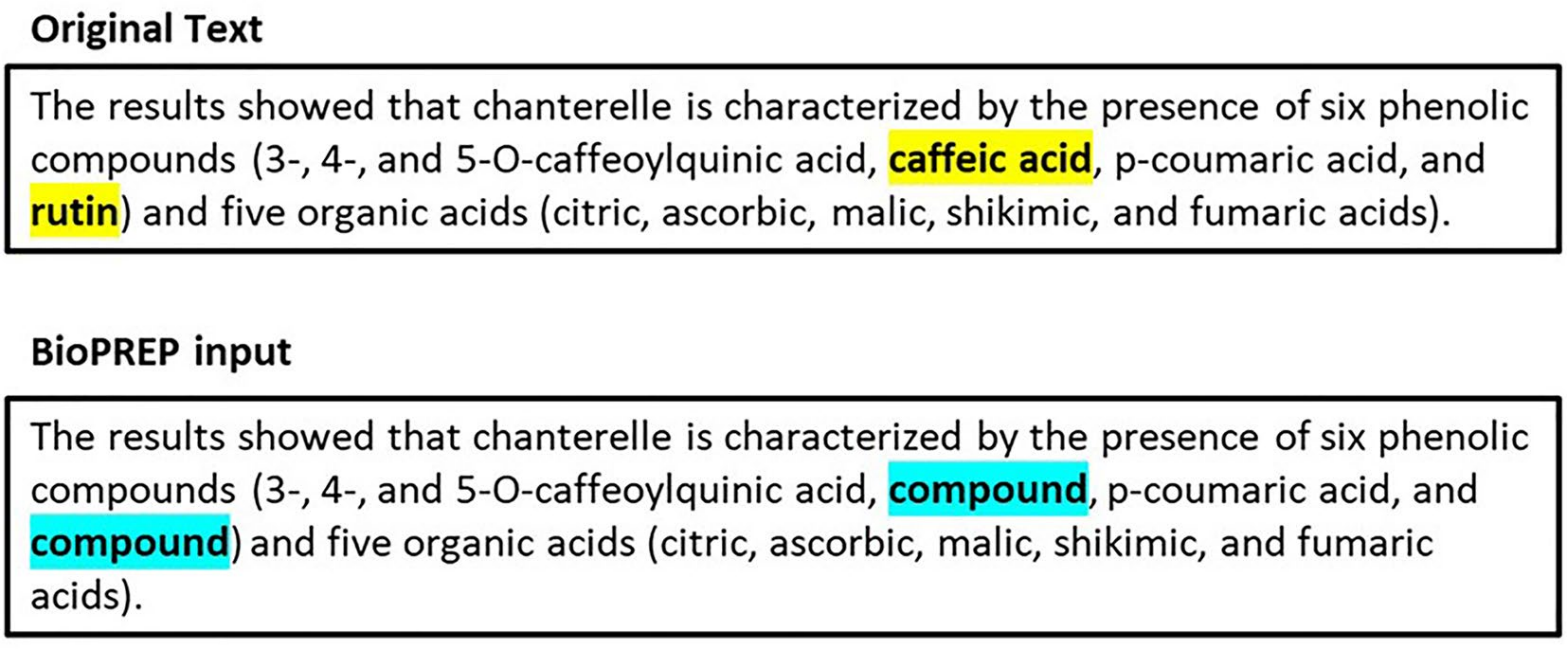
Pre-processing sentences by substituting entities with their type.

The pre-trained BioPREP model required entity type information for predicate classification. Hence, we needed to substitute entities with entity types before processing our sentences using the model, as illustrated in Fig. 2. If there were more than two unique bio entities in a sentence, we processed the entire sentence for relation extraction.

### Proposed path-ranking algorithm

After obtaining nodes and relations in the previous step, we built a knowledge graph and evaluated each possible path from Xanthium compounds to diabetes using our proposed path-ranking algorithm (PRA) framework. Our PRA framework consists of three steps: (1) transforming nodes and relations from the graph into vector representations (graph embedding). (2) Calculating triple (head node–relation–tail node) scores. The triples are bio entity pairs and relations obtained from the relation extraction step (“Relation extraction” section). (3) Calculating the path scores based on the average of triple scores and ranked paths accordingly. Paths with high scores have more inference possibilities, which might be necessary for constructing hypotheses.

Previous works in PRA employed co-occurrence and node similarity based on ontology to calculate the triple score (node–relation–node)^44^. However, using the co-occurrence number in PRA neglected the semantic relatedness between nodes because it ignores relation/edge type. Similar to co-occurrence, the previous approach in the triple score calculation using ontology information focused solely on the hierarchical positioning and neglected semantic relations between nodes^58^. These conditions might not be the best option for inference-purpose or hypothesis generation from path-ranking results. Therefore, this paper proposed a framework in PRA that includes relations to calculate the triple score.

Our framework employed a graph embedding approach called Complex^21^ to transform nodes and relations into vector representations (complex space). Previous research used Complex embeddings to execute link prediction tasks in knowledge graph completion^59^. Complex assumes a knowledge base as a three-way tensor to model asymmetric relations, matching relations in our knowledge graph. Complex decomposes tensor into low-dimensional vectors representing embedding values of entities and relations.

**Figure 3:**
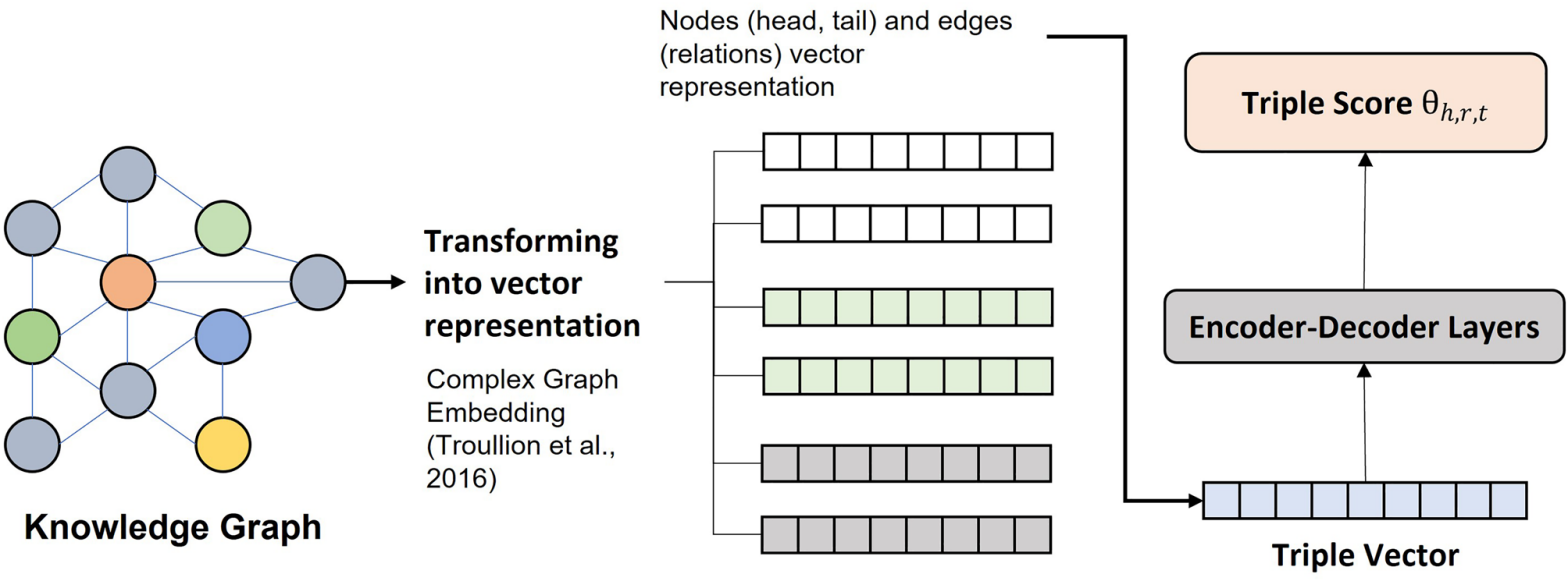
For calculating the triple score, we transform each node and edge into vector representation and construct triple vectors. Then, using the encoder–decoder architecture, we automatically generate weight values for the triple score calculation.

First, we trained our knowledge graph using the complex embeddings model and obtained the vector representation for nodes and relations. Then, we concatenated the head node, relation, and tail node vectors and constructed triple vectors. Later, we used the triple vectors as inputs for encoder–decoder architecture to obtain the weight values to calculate triple scores, as illustrated in Fig. 3. Later, we will use the weight values to transform the n-dimension vector into a probability that represents the triple score.

Our encoder–decoder architecture has seven layers. The first three are encoder layers, the fourth is the middle layer, and the last three are decoder layers. Our experiment only included the weight values from the last decoder layer as it encodes the latent representation of data. We used the mean-squared error loss in the training process to maintain the model correctness. We obtained the triple score by calculating the triple vector using Eq. (1). To rank paths, we calculated the path score of each path by averaging the total triple scores. For example, for paths with a depth of two (where there were two triples in the path), the path score would be the total of two triple scores divided by two.

1 $${\theta }_{h,r,t}={v}_{h,r,t}\cdot h$$

where n is the triple vector (v) dimension and h is the weight values obtained from the hidden layer.

### Hypothesis generation and evaluation

After executing the path-ranking algorithm, we conducted a thorough study of the biological linkages from the top-n paths. Our experts examined the top five percentile and concluded which paths were most plausible for drug development experiments. Furthermore, our experts examined paths in the middle and lower ranks to validate the performance of our proposed ranking algorithm.

## Result

### Xanthium compounds-diabetes knowledge base

The first step in knowledge base construction is entity extraction or NER task. We employed PKDE4J^19^ to process sentences and found that only 3,397,178 sentences contained bio entities. Initially, there were 145,246 unique bio entity terms; after normalization and disambiguation processes, only 84,176 bio entities remained. We provided a summary of NER task results in Table 4. Among 26,343 compounds, 144 compounds in total were related to *Xanthium*.

**Table 4:** NER result summary.

Entity type	Total
Phenotype	21,505
Compound	26,343
Gene	19,709
Protein	12,188
Biological Process	3414
Molecular Function	853
RNA	164
	84,176

We used the bio entity and entity type information obtained from the NER task to pre-process sentences for the second step, relation extraction. We should note that we only processed sentences with two or more entities and skipped sentences with only one entity. Table 5 gives the sample triples from relation extraction results. Similar node types might have more than one relation; for example, a phenotype can be a process of another phenotype or one phenotype can cause another phenotype, depending on the sentences registered as the BioPREP^20^ model input.

**Table 5:** Sample triples (head–relation–tail) from knowledge base.

	Head (type)	Relation	Tail (type)	Total
1	Phenotype	Process of	Phenotype	173,315
2	Phenotype	Coexists with	Phenotype	106,648
3	Phenotype	Is A	Phenotype	89,645
4	Compound	Is A	Compound	88,520
5	Compound	Coexists with	Compound	81,549
6	Gene	Coexists with	Gene	52,989
7	Protein	Coexists with	Protein	36,110
8	Phenotype	Associated with	Phenotype	34,761
9	Phenotype	Causes	Phenotype	27,918
10	Gene	Is A	Gene	27,464

We constructed a knowledge base using the obtained triple data from the relation extraction step. Then, we generated paths from 36 Xanthium compounds to five diabetes nodes (diabetes mellitus, type 1 diabetes, type 2 diabetes, gestational diabetes, and pre-diabetes). The generated paths were paths with a depth of two, three, and four. Unfortunately, we found no connecting paths between water-soluble glycosides and five diabetes nodes. This might be due to limited available information about water-soluble glycosides, as we only collected 14 related articles (as of January 2021). There are 12,437 paths with a depth of two, 3,612,585 with a depth of three, and 1,151,267,082 with a depth of four for 35 Xanthium compounds to five diabetes nodes.

**Figure 4:**
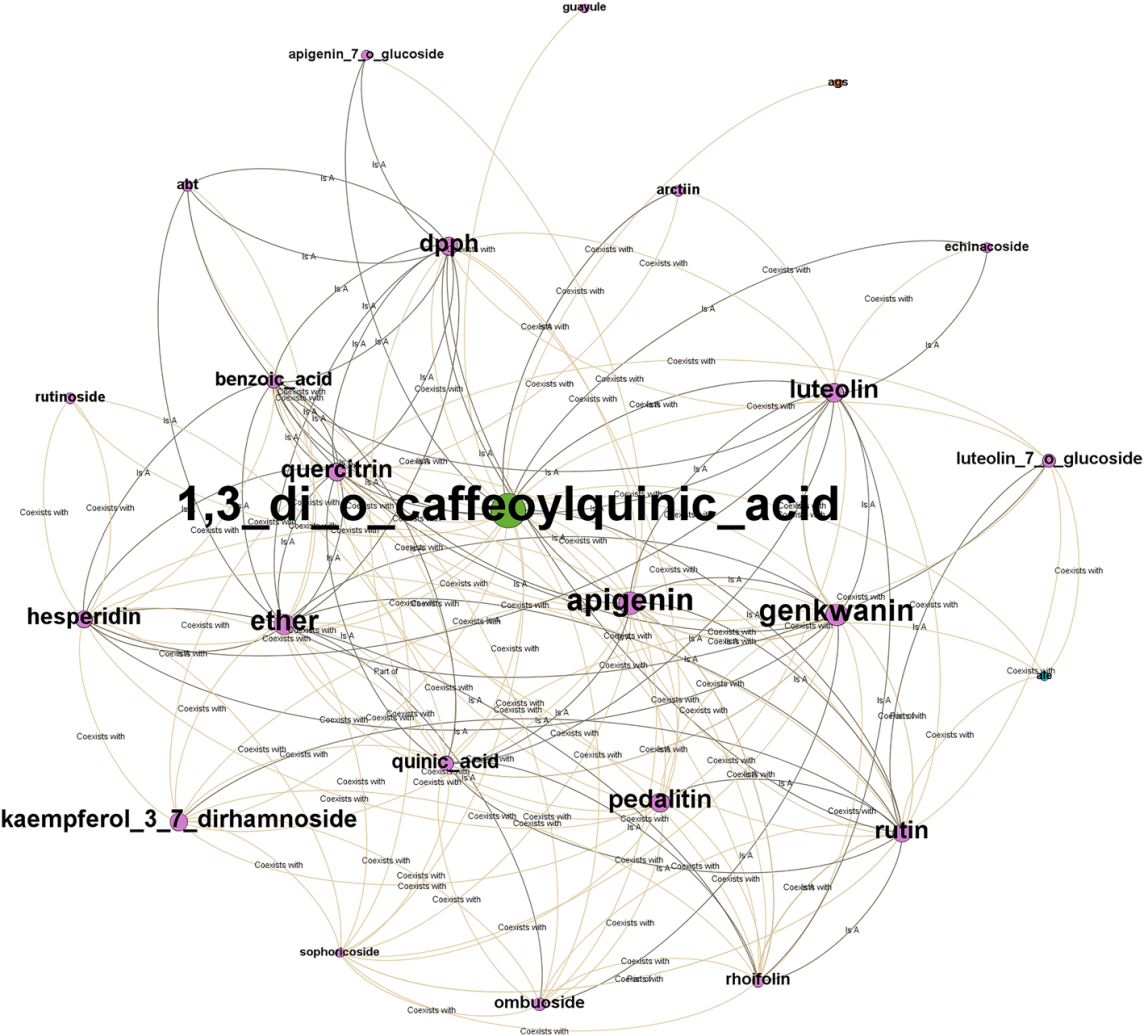
An ego graph of the compound “1,3_di_o_caffeoylquinic_acid” with radius = 1.

Given the large number of paths generated, we focused on “type 1 diabetes,” “type 2 diabetes,” and “diabetes mellitus” as tail nodes and a depth of two and three for further analysis. We provided the path summary between 35 Xanthium compounds and three diabetes-related phrases, “type 1 diabetes,” “type 2 diabetes,” and “diabetes mellitus,” in Table 6 and illustrated the subgraph of our knowledge base in Fig. 4. More paths were found from compounds like adenosine, choline, hexadecenoic acid, and quercetin than other compounds; these might indicate the high relatedness between those compounds and diabetes. We calculated scores for those paths and ranked them accordingly.

**Table 6:** Number of Paths for depth = 2 and depth = 3 from Xanthium compounds to “type 1 diabetes,” “type 2 diabetes,” and “diabetes mellitus.”

	Head	Total path					
		Type 1 diabetes		Type 2 diabetes		Diabetes mellitus	
		Depth 2	Depth 3	Depth 2	Depth 3	Depth 2	Depth 3
1	1,3_di_o_caffeoylquinic_acid	1	472	8	702	5	911
2	2_Acetolactate	3	1487	2	1732	4	2562
3	3,5_Dicaffeoylquinic_acid	2	260	2	360	4	457
4	4,5_Dicaffeoylquinic_acid	124	154	2	227	1	314
5	Acetone	336	38,651	198	46,892	289	66,230
6	Adenosine	177	80,372	436	95,341	708	134,650
7	Alkaloids	24	44,404	214	53,379	315	74,503
8	Aloe_emodin	9	9223	34	11,579	43	16,486
9	Atractyloside	1	5362	17	6497	17	9626
10	Balanophonin	35	755	5	1019	3	1390
11	Beta_sitostenone	38	409	4	607	3	767
12	Beta_sitosterol	47	16,384	52	21,073	80	29,085
13	Betulin	81	12,737	58	15,910	71	21,839
14	betulinic_acid	3	13,209	50	16,734	75	23,209
15	Caffeic_acid	22	28,589	115	36,282	181	50,355
16	Caffeic_acid_ethyl_ester	108	1155	9	1551	8	2170
17	Campesterol	190	8851	31	11,383	27	15,582
18	Chlorogenic_acid	69	35,819	147	44,429	214	61,171
19	Choline	59	54,070	290	64,827	448	90,269
20	emodin	90	20,301	94	24,973	145	34,885
21	Ergosterol	28	18,850	73	23,359	93	32,819
22	Ferulic_acid	190	30,105	127	37,381	166	51,512
23	Formononetin	1	9,422	33	11,991	39	17,150
24	Hexadecanoic_acid	56	60,202	253	73,441	338	101,224
25	n_trans_feruloyl_tyramine	169	576	4	792	3	1068
26	Oleanolic_acid	7	18,355	73	23,555	90	32,520
27	Oleic_acid	31	45,470	201	54,731	307	76,246
28	Ononin	215	1742	8	2121	7	2928
29	Protocatechuic_acid	61	12,071	44	15,650	63	21,602
30	Quercetin	24	57,706	297	70,084	462	97,745
31	Rhamnose	37	19,511	68	23,811	111	34,430
32	Scopoletin	5	8654	38	11,136	47	15,168
33	Stigmasterol	67	10,490	43	13,298	63	18,218
34	Syringaresinol	1	1646	10	2287	10	3037
35	Thiourea	3	25,694	90	31,105	146	44,759
	Total	2314	693,158	3130	850,239	4586	1,186,887

We can find the 35 compounds mentioned in Table 6 in the root, leaf, fruit, and aerial parts of Xanthium plants^60^. Despite findings of syringaresinol as a potential therapeutic agent for diabetes as it indicates the inhibition of inflammation, fibrosis, and oxidative stress^61^, we found fewer paths connecting the compound to diabetes. Similar to syringaresinol, there were relatively few paths for atractyloside and formononetin regardless of their significant relatedness with type 2 diabetes progression. Meanwhile, for compounds with evidence from a laboratory—such as beta-sitosterol and emodin—we found an adequate number of paths connecting those compounds to diabetes.

### Path-ranking performance evaluation

To ensure the performance of our proposed path-ranking algorithm, we conducted separate experiments using the Hetionet dataset. Hetionet is a bio entity network built using 29 publicly available databases containing 24 entity types (compounds, diseases, genes, biological pathways, etc.). Hetionet (version 1.0) contains 2,250,197 edges with 47,031 nodes from 11 types of bio entities. Although we can consider Hetionet a complete biological network (given how many datasets were integrated), it has little information regarding Xanthium compounds. A previous project called Repethio^62^ used Hetionet to identify paths from compound to disease and discriminate between treatments and non-treatments. The Repethio project gives a clear idea of how network-based data analysis significantly impacts drug development^63^.

The Repethio project predicted the probability of treatment for 209,168 compound–disease pairs (het.io/repurpose) and used two external sets of treatment for validation. This was an open study that received real-time evaluations from community members. For compound–disease prediction, they also provided network support analysis with information about path score and meta path contributions (meta path significance rate in treatment prediction). They calculated path scores using residual degree weighted path count (R-DWPC), a modification of the DWPC method introduced in^64^. Unlike the previous DWPC method, R-DWPC reflects the specific relationship between source and target nodes in paths. By assuming that the path score represents the level of significance (the higher, the better), we can also use the path score provided in the Repethio project for path ranking.

To validate our path-ranking algorithm, we used extracted paths between diabetes-related compounds and type 2 diabetes mellitus from Hetionet and compared ranking results based on path score with our path-ranking results. We retrieved paths with different depths: one, two, and three. We did not retrieve paths with a depth larger than four because Hetionet only provides information on path scores for paths with a depth of three or less. The compounds we used as source nodes for path retrieval were: Glyburide, Glipizide, Gemfibrozil, Tolazamide, Tolbutamide, Glimepiride, Telmisartan, Chlorpropamide, Losartan, Irbesartan, Eprosartan, Valsartan, Alogliptin, Nateglinide, Olmesartan, Gliclazide, Rosiglitazone, Methylergometrine, Repaglinide, and Fenofibrate. These compounds are recommended for diabetes disease^56^ and have a high probability of type 2 diabetes mellitus treatment according to Hetionet.

Using 20 compounds as source nodes and type 2 diabetes mellitus as the target node, we retrieved 13 paths with depth one, 132 paths with depth two, and 26,194 paths with depth three. For ranking results comparison, we employed rank-biased overlap (RBO)^65^ to calculate the similarity degree between our results (from PRA ranking) and Hetionet ranking. Table 7 shows the similarity degree based on RBO calculation for 20 compounds (with paths of depth two). In addition, we provided samples for path-ranking results with a depth of two for Glyburide to type 2 diabetes mellitus in Table 8. We provided their path-ranking results in additional material for other essential compounds.

**Table 7:** Similarity degree between our PRA and Hetionet ranking based on RBO analysis.

	Compounds	Depth	Total paths from hetionet	Similarity degree
1	Chlorpropamide	2	8	49.50%
2	Valsartan	2	5	53.30%
3	Glimepiride	2	7	53.80%
4	Tolazamide	2	7	53.80%
5	Tolbutamide	2	7	53.80%
6	Gliclazide	2	11	55.60%
7	Glipizide	2	11	59.60%
8	Olmesartan	2	6	59.70%
9	Eprosartan	2	3	66.70%
10	Nateglinide	2	6	69.40%
11	Glyburide	2	14	71.10%
12	Rosiglitazone	2	7	76.90%
13	Irbesartan	2	7	78.60%
14	Telmisartan	2	5	80.00%
15	Losartan	2	8	81.30%
16	Fenofibrate	2	3	83.30%
17	Gemfibrozil	2	5	83.30%
18	Repaglinide	2	5	93.30%
19	**Methylergometrine**	**2**	**4**	**100.00%**
20	**Alogliptin**	**2**	**3**	**100.00%**

Significant values are in bold.

**Table 8:** Ranking results for paths between Glyburide-type 2 diabetes mellitus with a depth of two.

Path	Our PRA Ranking	Hetionet Ranking
Glyburide—[treats]—gestational diabetes—[resembles]—type 2 diabetes mellitus	1	1
Glyburide—[resembles]—Tolazamide—[treats]—type 2 diabetes mellitus	2	3
Glyburide—[binds]—ABCC8—[associates]—type 2 diabetes mellitus	3	8
Glyburide—[binds]—KCNJ11—[associates]—type 2 diabetes mellitus	4	9
Glyburide—[resembles]—Glipizide—[treats]—type 2 diabetes mellitus	5	5
Glyburide—[resembles]—Chlorpropamide]—[treats—type 2 diabetes mellitus	6	6
Glyburide—[resembles]—Glimepiride—[treats]—type 2 diabetes mellitus	7	2
Glyburide—[binds]—ABCC2—[associates]—type 2 diabetes mellitus	8	10
Glyburide—[resembles]—Gliclazide—[treats]—type 2 diabetes mellitus	9	4
Glyburide—[binds]—CPT1A—[associates]—type 2 diabetes mellitus	10	7
Glyburide—[binds]—CYP3A4—[associates]—type 2 diabetes mellitus	11	11
Glyburide—[binds]—ALB—[associates]—type 2 diabetes mellitus	12	12
Glyburide—[downregulates]—VEGFA—[associates]—type 2 diabetes mellitus	13	14
Glyburide—[downregulates]—HIF1A—[associates]—type 2 diabetes mellitus	14	13

For paths with depth two, the similarity was in the range 49.5–100% with an average value of 71.2%; the Alogliptin and Methylergometrine paths reached 100% similarity. The average similarity degree for paths with a depth of three was slightly lower as the number of paths was increased. The average value was 49.8% and the range was 44.5–54.7%. We should note that our proposed approaches in path scoring and Hetionet differ considerably. Hetionet weighted each path (edge) by calculating node degrees’ product and raising it to a negative exponent. Meanwhile, our approach focused on weighting each path using graph embedding values translated from complex space.

### Path-ranking results (Xanthium compounds—diabetes)

There were 2,740,314 paths between 35 Xanthium compounds and the three diabetes nodes type 1 diabetes, type 2 diabetes, and diabetes mellitus. We calculated the path score by averaging the triple scores obtained from Eq. (1). Then, we sorted those paths and analyzed paths in the top five percentile. There were 34,774 paths for type 1 diabetes, 42,670 for type 2 diabetes, and 59,575 for diabetes mellitus. Among the top-ranked paths linked to type 1 diabetes, compounds such as adenosine, alkaloids, quercetin, choline, and oleic acid were dominant. For type 2 diabetes, based on the number of occurrences in top percentile paths, the most significant compounds were adenosine, quercetin, alkaloids, choline, and caffeic acid. Lastly, for diabetes mellitus, the most significant compounds were adenosine, quercetin, alkaloids, choline, and hexadecenoic acid. Table 9 provides the top ten paths of each diabetes term.

**Table 9:** Top ten paths of Xanthium compounds—three diabetes terms.

Rank	Path	Score
*Type 1 diabetes*		
1	beta_sitosterol—[Stimulates]—glucose—[Associated with]—type_1_diabetes	0.822
2	rhamnose—[Associated with]—glucose—[Associated with]—type_1_diabetes	0.820
3	adenosine—[Associated with]—labetalol—[Associated with]—glucose—[Associated with]—type_1_diabetes	0.819
4	alkaloids—[Parts of]—aucubin—[Treats]—glucose—[Associated with]—type_1_diabetes	0.819
5	adenosine—[Coexists with]—allicin—[Affects]—glucose—[Associated with]—type_1_diabetes	0.818
6	scopoletin—[Stimulates]—glucose—[Associated with]—type_1_diabetes	0.818
7	alkaloids—[Associated with]—phenytoin—[Causes]—glucose—[Associated with]—type_1_diabetes	0.818
8	beta_sitosterol—[Stimulates]—glucose—[Associated with]—methanol—[Neg Affects]—type_1_diabetes	0.818
9	oleic_acid—[Neg Affects]—gallic_acid—[Stimulates]—glucose—[Associated with]—type_1_diabetes	0.817
10	rhamnose—[Associated with]—glucose—[Associated with]—methanol—[Associated with]—type_1_diabetes	0.817
	*Type 2 diabetes*	
1	alkaloids—[Administered to]—diabetic_complication—[Associated with]—autoimmune_disease—[Associated with]—type_2_diabetes	0.824
2	alkaloids—[Administered to]—diabetic_complication—[Causes]—arthritis—[Associated with]—type_2_diabetes	0.823
3	alkaloids—[Administered to]—diabetic_complication—[Associated with]—autoimmune_disease—[Associated with]—type_2_diabetes	0.822
4	alkaloids—[Administered to]—diabetic_complication—[Causes]—vasculitis—[Associated with]—type_2_diabetes	0.822
5	quercetin—[Coexists with]—diabetic_complication—[Associated with]—autoimmune_disease—[Associated with]—type_2_diabetes	0.821
6	alkaloids—[Administered to]—diabetic_complication—[Causes]—neurological_disorder—[Associated with]—type_2_diabetes	0.821
7	alkaloids—[Administered to]—diabetic_complication—[Associated with]—chronic_lung_disease—[Associated with]—type_2_diabetes	0.820
8	alkaloids—[Administered to]—diabetic_complication—[Causes]—optic_neuropathy—[Associated with]—type_2_diabetes	0.820
9	quercetin—[Coexists with]—diabetic_complication—[Causes]—arthritis—[Associated with]—type_2_diabetes	0.820
10	alkaloids—[Administered to]—diabetic_complication—[Associated with]—hypokalemia—[Associated with]—type_2_diabetes	0.820
*Diabetes Mellitus*		
1	adenosine—[Treats]—congestive_heart_failure—[Associated with]—diabetes_mellitus	0.853
2	adenosine—[Treats]—asthma—[Associated with]—diabetes_mellitus	0.852
3	adenosine—[Neg Affects]—luteolin—[Stimulates]—diabetes_mellitus	0.850
4	alkaloids—[Causes]—hyperlipidemia—[Associated with]—diabetes_mellitus	0.850
5	adenosine—[Treats]—lymphoma—[Associated with]—diabetes_mellitus	0.850
6	adenosine—[Associated with]—inflammatory_bowel_disease—[Associated with]—diabetes_mellitus	0.849
7	alkaloids—[Treats]—heart_disease—[Associated with]—diabetes_mellitus	0.849
8	alkaloids—[Associated with]—autoimmune_disease—[Associated with]—diabetes_mellitus	0.848
9	adenosine—[Treats]—pulmonary_disease—[Associated with]—diabetes_mellitus	0.847
10	alkaloids—[Treats]—metabolic_disease—[Associated with]—diabetes_mellitus	0.847

Based on the top percentile paths, diabetes is strongly related to adenosine, alkaloids, choline, and quercetin. As reported in the clinical trials sections from the Drug Bank^56^, some records stated that adenosine and choline are related to diabetes. Adenosine was used for diabetes mellitus and type 2 diabetes experiments but there was no further information about the clinical trial phase or purpose. However, a record mentioned that a clinical experiment for diabetic peripheral neuropathic pain treatment had entered phase four of the clinical trial for choline. In addition, another clinical experiment used choline for type 2 diabetes mellitus treatment and entered the third phase of clinical trials.

Alkaloids are natural chemical compounds derived from plants, animals, bacteria, or fungi with various pharmacological activities^17^. Naturally, derived alkaloids were effective for diabetic nephropathy treatments and suitable for patients who did not respond well to synthetic drugs or conventional therapeutic medications^66^. Alkaloids could be a strong candidate for the new discovery of anti-diabetic agents. In addition to alkaloids, quercetin might be a potential candidate for diabetes treatment. Quercetin is one of the plant-based flavonoids with various potent biological properties including anti-inflammatory, antioxidative, anti-hypertensive, anticancer, antiviral, neuroprotective, hepatoprotective, and anti-diabetic^67^. Although there has not been any clinical trial record of quercetin for diabetes treatment, there was a completed phase one clinical trial for quercetin as a treatment purpose in high blood pressure disease (hypertension). Previous work mentioned that diabetes patients with hypertension were more predisposed to several complications^68^.

In addition to adenosine, alkaloids, choline, and quercetin, we discovered that caffeic acid, hexadecenoic acid, and oleic acid were also significantly related to diabetes. Those acids are essential to maintaining the diabetes patients’ diets. Caffeic acid could suppress the progression of type 2 diabetes states^69^. High hexadecenoic acid or palmitoleic acid in diets were highly associated with higher risks of diabetes^70^. Lastly, oleic acid helped prevent type 2 diabetes and cardiovascular diseases^71^. According to clinical trial records (as of January 2021), among 36 Xanthium compounds, only two—adenosine and choline—have been reportedly used for diabetes clinical trials. Those two compounds were also in the top selection based on our PRA results. After matching findings from top percentile paths with previous research—including clinical trials—we concluded that our PRA framework distinguished critical paths for hypothesis generation.

### Hypothesis generation

Our experts analyzed the top-ranked paths (the top five percentile) and compiled information for Xanthium compounds and diabetes. Previous research showed significant relationships between diabetes and adenosine, oleic acid, choline, caffeic acid, and stigmasterol. From the constructed Xanthium compounds and diabetes, there was a direct edge between those compounds and diabetes. In addition, several paths with a depth of two or three connected those compounds and diabetes diseases. Based on those paths, we concluded that choline and betaine intake were supplementary to type 2 diabetes^72^. Caffeic acid has antioxidant properties that might prevent several chronic diseases including diabetes^73^. Moreover, stigmasterol had the potential to protect beta cell functions during diabetes progression^74^. Other compounds were also connected to diabetes disease through intermediary nodes that are most likely to accelerate diabetes progression, such as hypertension and infections.

The type 1 diabetes-related paths showed significant relatedness between several Xanthium compounds and glucose. Glucose is the main compound in carbohydrate metabolism and provides energy by ATP synthesis. Cells in diabetic patients cannot process glucose effectively due to insulin decrease, resulting in a high glucose level. Compounds such as adenosine, beta-sitosterol, rhamnose, and scopoletin could show decreased glucose level. Based on the data in our collection, we found 2808 documents supporting the argument about adenosine, glucose, and diabetes. For others, we found 73 articles on beta-sitosterol, 63 articles on rhamnose, and 12 articles on scopoletin. Based on those numbers, we can assume that researchers had explored adenosine and diabetes further but only a few had shown interest in the other three compounds. These three compounds might be more appropriate selections for hypothesis generation results than adenosine. Since there were only a few publications related to those compounds and diabetes, we believe that there might be more discoveries to be made; we strongly recommend them for further experiments concerning the glucose level in diabetes cases.

Based on the top percentile paths, we found that adenosine had a significant role in diabetes prognosis. Adenosine is an agonist of adenosine receptors with binding functions that trigger biological reactions. Adenosine receptor signaling plays an essential role in inflammation, immune systems, and oxidative stress^75^. Thus, adenosine was highly related to heart disease, ischemic heart disease, autoimmune disease, and lymphoma. Those diseases are metabolic syndromes related to diabetes. Since we only observed paths with a depth of two and three, the intermediary nodes (between Xanthium compound and diabetes-related terms) were mostly compound or disease nodes. Therefore, for further experiments with more variations in intermediary nodes, we recommended using paths with a depth more extensive than three.

Based on our findings about the top five percentile paths, we concluded the following hypotheses.

- Compounds that negatively affect glucose level (lowering effect) are potential candidates for diabetes drug development.
- Compounds that are beneficial to treat diseases related to higher diabetes risks are potential candidates for diabetes drug development.
- We recommended adenosine, choline, beta-sitosterol, rhamnose, and scopoletin for further studies in diabetes drug development.

## Conclusions

Previous hypothesis-generation approaches depended on how experts summarized published scientific documents or how experts interpreted knowledge bases. Similar to previous approaches, we experimented with published scientific documents and expert judgments to generate hypotheses for diabetes drug development using compounds from *Xanthium*. Our hypothesis generation framework used evidence from scientific publications retrieved from PubMed to build a Xanthium compounds-diabetes knowledge base and generate hypotheses from it. First, we employed a dictionary-based tool to conduct the NER task and extracted bio-entities such as genes, compounds, phenotypes, biological processes, and molecular functions. Depending on the size and coverage of dictionaries, using a dictionary-based tool might be beneficial for recognizing bio-entities. Second, we classified possible relations between two entities using entity type information obtained from the NER task step and analyzed sentences’ context. We trained sentences where two entities were found in a supervised manner using a deep learning approach. The relation classification step gave us triple information (node–relation–node), which enabled us to construct a knowledge base.

Using the constructed knowledge base, we generated simple paths from Xanthium compounds to three diabetes-related phrases: type 1 diabetes, type 2 diabetes, and diabetes mellitus. We used several cutoffs to generate paths and analyzed paths with depths of two and three, which we then ranked using our proposed PRA. Our proposed PRA approach utilized a graph-embedding model to transform nodes and relations (edges) into vector representations. Then, we constructed the triple (node–relation–node) vector representation by concatenating individual vectors and used them to calculate the triple score. Lastly, we calculated the path score based on the average of total triple scores in a path. We considered paths with high path scores as significant paths that might be helpful for hypothesis generation. Using PRA, we made shortlists of important information from an extensive knowledge base. In addition, this helped our experts generate hypotheses related to Xanthium compounds and diabetes. Since our proposed PRA approach employed graph embedding, the results depended on how well the graph was constructed. A larger graph with complete information might give better results than smaller ones. Unfortunately, we only experimented with one graph embedding algorithm in this research, Complex. We plan to do more comprehensive experiments with other graph-embedding algorithms for further analysis.

## Supplementary Information

Supplementary Information 1.

Supplementary Information 2.

## Data Availability

The datasets used and/or analyzed during the current study are available from the corresponding author upon reasonable request.
