# Supplementary material for: An automatic hypothesis generation for plausible linkage between xanthium and diabetes
Source: Sci Rep. 2022 Oct 20;12:17547. doi: 10.1038/s41598-022-20752-0 (PMC9585073; doi:10.1038/s41598-022-20752-0)
Supplement: Supplementary file 1 — Supplementary Information 1. [file 41598_2022_20752_MOESM1_ESM.docx]

# ADDITIONAL MATERIAL

- Additional File 1: Path-ranking Performance Evaluation

The comparison between path-ranking results from proposed PRA and Hetionet scoring with six columns: number, head node (20 compounds related to diabetes), paths, depth of path, ranking from proposed PRA, and ranking based on Hetionet scoring.
